# Alternative Quantifications of Landscape Complementation to Model Gene Flow in Banded Longhorn Beetles [*Typocerus v. velutinus* (Olivier)]

**DOI:** 10.3389/fgene.2020.00307

**Published:** 2020-03-31

**Authors:** Richard Borthwick, Alida de Flamingh, Maximilian H. K. Hesselbarth, Anjana Parandhaman, Helene H. Wagner, Hossam E. M. Abdel Moniem

**Affiliations:** ^1^Department of Biological and Environmental Sciences, Alabama A&M University, Normal, AL, United States; ^2^Program in Ecology, Evolution, and Conservation Biology, University of Illinois at Urbana–Champaign, Urbana, IL, United States; ^3^Department of Ecosystem Modelling, University of Göttingen, Göttingen, Germany; ^4^Department of Geography, Program in Ecology, Evolution, and Conservation Biology, University of Nevada, Reno, Reno, NV, United States; ^5^Department of Ecology & Evolutionary Biology, University of Toronto, Mississauga, ON, Canada; ^6^Department of Zoology, Suez Canal University, Ismailia, Egypt; ^7^Centre for Urban Environments, University of Toronto, Mississauga, ON, Canada

**Keywords:** landscape configuration, maximum-likelihood population effects, surface metrics, landscape metrics, gradient surface model, patch mosaic model, isolation-by-resistance, complementary habitat

## Abstract

Rapid progression of human socio-economic activities has altered the structure and function of natural landscapes. Species that rely on multiple, complementary habitat types (i.e., landscape complementation) to complete their life cycle may be especially at risk. However, such landscape complementation has received little attention in the context of landscape connectivity modeling. A previous study on flower longhorn beetles (*Cerambycidae*: *Lepturinae*) integrated landscape complementation into a continuous habitat suitability ‘surface’, which was then used to quantify landscape connectivity between pairs of sampling sites using gradient-surface metrics. This connectivity model was validated with molecular genetic data collected for the banded longhorn beetle (*Typocerus v. velutinus*) in Indiana, United States. However, this approach has not been compared to alternative models in a landscape genetics context. Here, we used a discrete land use/land cover map to calculate landscape metrics related to landscape complementation based on a patch mosaic model (PMM) as an alternative to the previously published, continuous habitat suitability model (HSM). We evaluated the HSM surface with gradient surface metrics (GSM) and with two resistance-based models (RBM) based on least cost path (LCP) and commute distance (CD), in addition to an isolation-by-distance (IBD) model based on Euclidean distance. We compared the ability of these competing models of connectivity to explain pairwise genetic distances (*R*_ST_) previously calculated from ten microsatellite genotypes of 454 beetles collected from 17 sites across Indiana, United States. Model selection with maximum likelihood population effects (MLPE) models found that GSM were most effective at explaining pairwise genetic distances as a proxy for gene flow across the landscape, followed by the landscape metrics calculated from the PMM, whereas the LCP model performed worse than both the CD and the isolation by distance model. We argue that the analysis of a continuous HSM with GSM might perform better because of their combined ability to effectively represent and quantify the continuous degree of landscape complementation (i.e., availability of complementary habitats in vicinity) found at and in-between sites, on which these beetles depend. Our findings may inform future studies that seek to model habitat connectivity in complex heterogeneous landscapes as natural habitats continue to become more fragmented in the Anthropocene.

## Introduction

Rapid progression of landscape changes associated with human socio-economic activities in the Anthropocene alters the structure and function of natural landscapes (DeFries et al., 2004; Lee et al., 2006; Hobbs et al., 2009). One such direct impact is the transformation of ecosystem edges and ecotones (transition zones between ecosystems) (Fortin et al., 2000; Stewart et al., 2013), which may disturb nutrient and phenological cycles (Décamps and Naiman, 1990) of different taxa. For instance, land-use change and disturbance have threatened functional diversity in terrestrial arthropods (Birkhofer et al., 2015), economic and agricultural development has impacted microbial ecosystems and communities (Pepper et al., 2015), contributed to landscape erosion and aridification (Zerboni and Nicoll, 2019), and altered ecosystem function through shifts in key phenological events like flowering (Calinger, 2015). At the landscape level, anthropogenic impacts have been acknowledged among main drivers of habitat loss and fragmentation leading to habitat isolation, and increasing barriers to movement of organisms and their genes across the landscape (Fortin et al., 2000; Lindenmayer and Fischer, 2007). In order to better manage landscapes that support human socio-economic development and maintain natural habitats that support biodiversity, we need an effective and accurate quantification of landscape heterogeneity and connectivity at biologically meaningful scales.

Landscape genetics provides explicit methods for quantifying the effects of landscape spatial heterogeneity on gene flow and spatial genetic structure of organisms (Storfer et al., 2007). Historically, landscape genetic studies have been constrained by cost-prohibitive genetic data collection (Shendure and Aiden, 2012), limited landscape-scale habitat information, and difficulties in characterizing spatial heterogeneity at a meaningful scale and linking that heterogeneity to genetic patterns. However, advances in DNA isolation techniques (e.g., Davey et al., 2011), reductions in genetic data-collection costs (Shendure and Aiden, 2012), and increasing access to large landscape-level datasets [e.g., the National Land Cover Database (NLCD) in the United States or large-scale digital elevation models] have resulted in lower costs and increased data availability. Therefore, finding optimal modeling approaches and metrics to represent the environment and assess genetic patterns of species across the landscape has become a new challenge.

**FIGURE 1:**
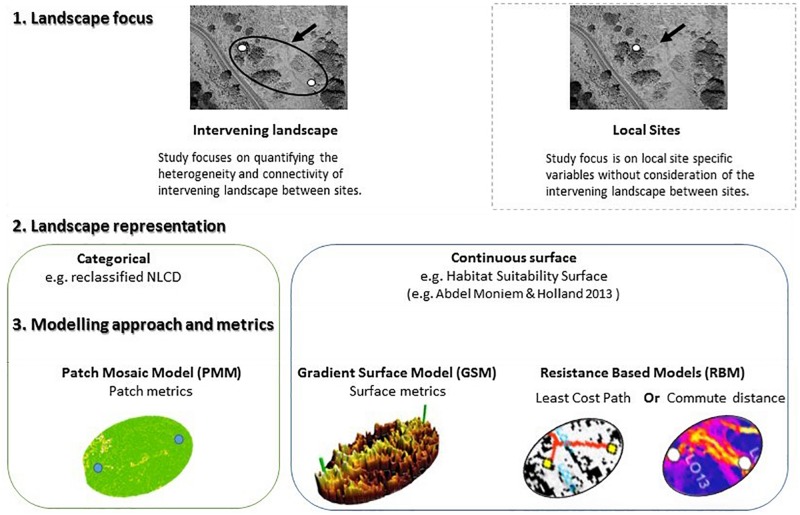
Conceptual framework of various landscape modeling approaches in landscape genetics illustrating the difference between study design focus (site specific vs. landscape), and modeling approaches (categorical vs. continuous) representation of habitat in the landscape.

Capturing biologically relevant landscape features for building a reliable connectivity model is a challenging task that depends on the species’ distribution and life history. This task may be particularly challenging in species that require landscape complementation, where different life history stages rely on multiple, complementary habitat types (e.g., Dunning et al., 1992; Pope et al., 2000; Mandelik et al., 2012). While landscape complementation has received little attention in the context of landscape connectivity modeling (and vice versa) it provides a unique context to examine the ability of different conceptualizations of landscape heterogeneity and connectivity modeling approaches to explain genetic patterns across a landscape. In this study we compare the ability of fundamentally different descriptors of habitat heterogeneity (Figure 1) to explain genetic dissimilarities among populations of a species reliant on landscape complementation – the banded longhorn beetle [*Typocerus v. velutinus* (Olivier)].

Banded longhorn beetles require multiple phenological habitats throughout their life-history that can only be supported by landscape complementation. The species’ requirements for completing its life cycle consist of open flowering habitat (shrublands, grasslands, or pastures), which is required for adults’ feeding and mating, adjacent to forested woodlands that contain dead and decaying wood, which supports larval development (Linsley, 1959).

To explain pairwise genetic distances as a proxy of gene flow between sampling locations (sites), we evaluated landscape heterogeneity and connectivity using discrete and continuous landscape representations. For the discrete landscape representation, we used a categorical land use/land cover map (NLCD 2011; Homer et al., 2015) to calculate landscape metrics based on the PMM. The PMM has routinely been used to describe landscape composition (amount of each habitat type) and configuration (spatial arrangement of habitat types) (e.g., Pringle et al., 1988; Carrara et al., 2015). This approach continues to have applicability (Fourcade et al., 2017) due to its conceptual simplicity and consistency with statistical analysis frameworks (e.g., ANOVA) (McGarigal et al., 2009). However, PMM has been criticized for its over-simplicity, and lacking the capacity to describe the continuous nature of habitat heterogeneity in natural landscapes (McGarigal et al., 2009).

A few early papers represented landscapes as gradients and not as discrete land use classes (Jeltsch et al., 1970; McIntyre and Barrett, 1992; Manning et al., 2004; Lindenmayer and Fischer, 2007), and this approach has become more widely applied in recent years (e.g., McGarigal et al., 2009; Abdel Moniem and Holland, 2013). A gradient approach is directly applicable in many natural landscapes that are dominated by gradual changes, whereas many agricultural landscapes are compatible with a PMM due to anthropogenic patterns of land use that create discrete patches. Abdel Moniem and Holland (2013) modeled habitat suitability for flower visiting longhorn beetles (*Cerambycidae*: *Lepturinae*) that require landscape complementation by evaluating discrete and continuous landscape features simultaneously in a moving-window analysis. This resulted in a continuous HSM that can be analyzed as a gradient surface. The HSM can thus represent complex landscape characteristics (e.g., Abdel Moniem and Holland, 2013), including the nearby availability of complementary habitat types.

Here, we used the HSM previously derived by Abdel Moniem and Holland (2013) that describes gradients in habitat suitability for flower-visiting longhorn beetles as a continuous surface. The model considered habitat complementarity by incorporating discrete (e.g., NLCD), and continuous (e.g., digital elevation models or DEM, curvature index, solar insolation, NDVI, and splitting index) landscape variables (see Abdel Moniem and Holland, 2013 for more details). These variables represent aspects of habitat quality and the local availability of resources for both larval and adult development. Abdel Moniem and Holland (2013) analyzed the topology of this HSM to quantify landscape heterogeneity using GSM. These metrics describe different aspects of surface roughness, the shape of the surface height distributions, surface angular texture, and surface radial texture and magnitude (reviewed in McGarigal et al., 2009; Abdel Moniem et al., 2016). The GSM were found to be powerful descriptors of the continuous, undulating shape of habitat suitability surfaces and effective predictors of the community composition of longhorn beetles (Abdel Moniem and Holland, 2013).

In the landscape genetic literature, landscape connectivity is commonly assessed through resistance modeling based on the idea that different landscape features pose different levels of resistance to organism movement (Adriaensen et al., 2003; McRae, 2006). Resistance values, which are quantitative by nature, can be assigned to discrete land-use/land-cover classes (McRae et al., 2008). Alternatively, a resistance surface (or its inverse, conductance) can be derived by transforming a HSM model (Keeley et al., 2016). Either way, the resistance of the intervening landscape between any pair of sampling locations can then be evaluated either as a single-best corridor (LCP; Adriaensen et al., 2003), or as CD based on circuit theory (McRae, 2006; McRae et al., 2008; Shah and McRae, 2008), which considers all possible paths.

This study aimed to compare approaches of modeling functional connectivity for an organism that requires multiple, complementary habitat types to complete its life cycle, starting from discrete (PMM) and continuous (HSM) landscape representations (Figure 1). The PMM landscape was characterized by two different methods using landscape metrics related to landscape complementation. The HSM landscape representation published by Abdel Moniem and Holland (2013) was evaluated with GSM, and with LCP and CD as two alternative RBM. We then compared the ability of the different models, including a null model of IBD, to explain genetic distances for banded longhorn beetle among sampling sites, using the genetic data from Abdel Moniem et al. (2016). We thus aimed to identify which modeling approach best represented connectivity for banded longhorn beetle, a species that requires landscape complementation.

## Materials and Methods

### Study Area and Genetic Data

**FIGURE 2:**
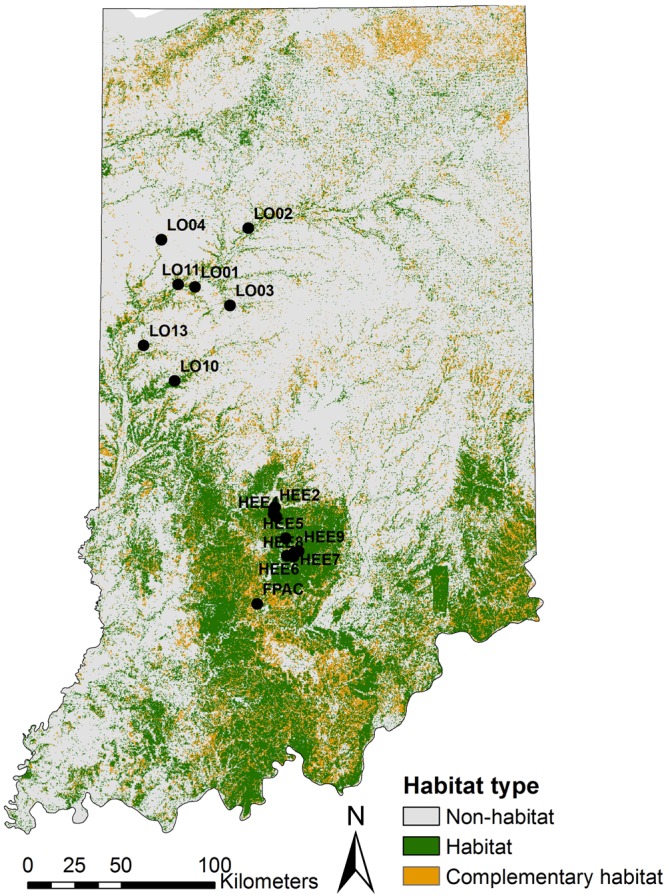
A map of Indiana showing locations of the study sites on a NLCD map. NLCD layer was reclassified into three classes of considered land cover; habitat (all forests), complementary habitat (shrublands, grasslands or pastures), and non-habitat (remaining land-cover classes). Black circles indicate sampling locations.

We re-analyzed genetic data from Abdel Moniem et al. (2016), which consisted of ten polymorphic microsatellite loci for 454 individuals sampled from 17 sampling locations across Indiana (United States; Figure 2) between 2005 and 2011. These microsatellite markers were specifically developed for longhorn beetles in the study area and were screened for genotyping errors and polymorphism prior to use [see Abdel Moniem et al. (2016) for a detailed description of the methods used for developing, screening, and amplifying these markers].

To delineate the local landscapes that individuals were most likely to encounter while dispersing between any pair of sampling locations, we used ellipsoids delineated by Abdel Moniem et al. (2016) based on a correlated random walk approach (Okubo and Kareiva, 2001; Koh et al., 2013). Successful correlated random walks (i.e., walks that started at sampling location *i* and were able to reach sampling location *j* using specific distributions of turning angle, step length, and total number of steps between sites separated from each other by various distances) were used to estimate the parameters of an ellipsoid connecting the two sampling locations. Using these parameters, we clipped a total of 136 ellipsoids connecting all possible pairs of sampling locations and quantified the heterogeneity within each of these local landscapes with the different approaches. As the response variable, we quantified pairwise genetic distance between sampling locations with an *R*_ST_ dissimilarity matrix, following Abdel Moniem et al. (2016). *R*_ST_ accounts for allele size differences by assuming a stepwise mutation model of marker evolution (Slatkin, 1995) and was consistently found to be a better proxy for gene flow for longhorn beetles than Wright’s *F*_ST_ (Abdel Moniem et al., 2016). However, because the choice of the genetic distance may influence the model outputs, we also tested proportion of shared alleles (*D*_PS_; Bowcock et al., 1994; Chapuis and Estoup, 2006) as a measure of genetic distance between sampling sites. *D*_PS_ relies on the infinite allele model (Nei et al., 1976) and was found to produce lower model fits based on *R*^2^ values, and resulted in slightly different landscape models’ structure and ranking (see Supplementary Material for *D*_PS_ results).

### Habitat Suitability Model and Gradient Surface Metrics

**FIGURE 3:**
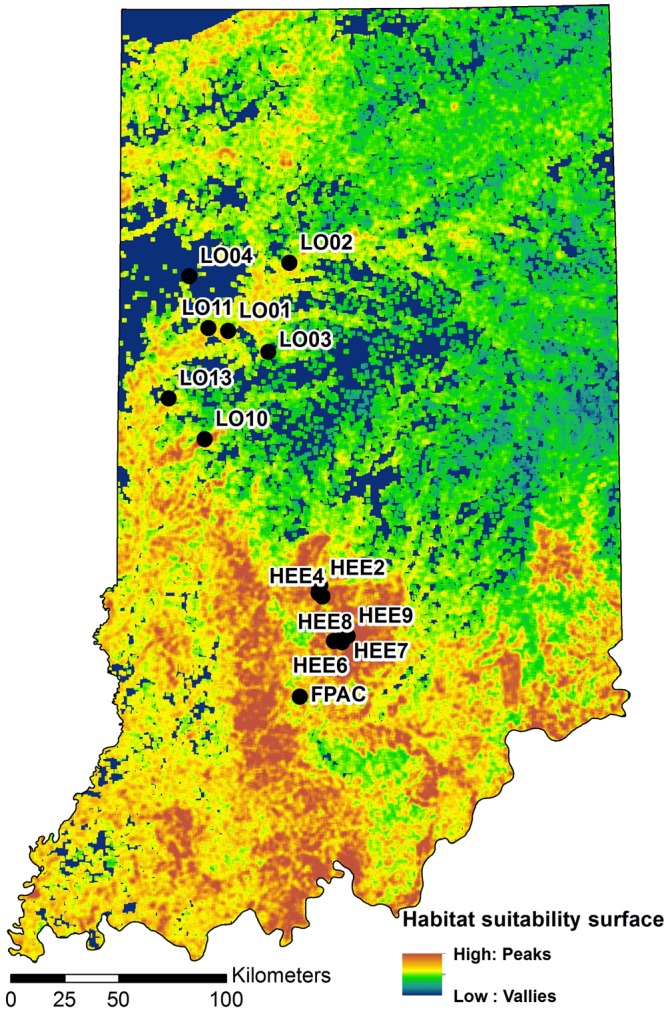
A map of Indiana showing locations of the study sites on a habitat suitability surface for the banded longhorn beetle (adopted from Abdel Moniem et al., 2016). Black circles indicate sampling locations.

We used the same habitat suitability model surface (HSM; Figure 3) as Abdel Moniem et al. (2016), which was derived through a moving-window analysis of six GIS layers that were chosen to represent both larval and adult biological and physical requirements: percentage forest, landscape splitting index, NDVI, digital elevation model, curvature index, and solar insolation. For each layer, the mean was calculated within a square moving window with 2.1 km edge length. The first three biological layers represent forest fragmentation and health and the latter three geophysical layers describe topography, slope, and insolation, which may also be important for the species (Abdel Moniem and Holland, 2013; Abdel Moniem et al., 2016). In this approach, complementarity was addressed by incorporating the percent forest and NDVI layers as descriptors of the primary habitat. Complementary habitat, which consists of open areas with flowering resources within and around forest, was accounted for with the splitting index, which is an aggregation metric that describes the degree of subdivision of the landscape (McGarigal et al., 2009).

Gradient surface metrics describe the continuous, undulating shape of habitat suitability surfaces, and have been shown to be informative in describing habitat and predicting gene flow across the landscape in this system (Abdel Moniem et al., 2016). Here, we recalculated 10 metrics that were previously described by Abdel Moniem et al. (2016) to characterize the HSM surface within each of the 136 ellipsoids (see Abdel Moniem et al., 2016 for a detailed description of individual metrics and the methods used to calculate them, and Table 1 for a brief description of each metric). We removed correlated metrics using the variance inflation factor until all metrics had a VIF < 10. Only one metric needed to be removed, resulting in nine remaining metrics (Table 1).

**TABLE 1:** Gradient surface metrics (GSM) to describe the landscape heterogeneity in the context of the habitat suitability model (HSM).

**Metric**	**Abbreviation**	**Aspect of landscape described**
Average surface roughness	*Sa*	(Non-spatial) landscape diversity
Ten-point height	*S10*	(Non-spatial) landscape richness
Skewness	*Ssk*	Skewness of habitat quality values (patch-based evenness)
Surface area ratio	*Sdr*	Ratio between surface area and flat plane (contrast-weighted edge density).
Dominant texture direction	*Std*	Direction of dominant amplitude (landscape composition and configuration).
Texture direction index	*Stdi*	Dominance relative to directions (variability in distribution and spatial arrangement of surface heights).
Radial wavelength index	*Srwi*	Wavelengths relative to all radial distances (sensitive to variability in surface heights).
Fractal dimension	*Sfd*	Angles of the angular spectrum based on Fourier analysis (landscape configuration)
Surface bearing index	*Sbi*	Measure of landscape dominance (Matrix and patch distribution in the landscape)

### Patch Mosaic Model and Landscape Metrics

In order to describe the landscape using the PMM, we re-classified the National Land Cover Database (NLCD 2011; Homer et al., 2015) into three different classes based on the habitat requirements of the banded longhorn beetle (Abdel Moniem and Holland, 2013) (Figure 2). We used data from the 2011 NLCD census since this is the land cover most likely experienced by the beetle populations sampled for this study. All forest-related land cover classes were classified as “forest habitat.” Shrublands, grasslands and pastures in the NLCD were classified as “complementary habitat,” whereas all remaining land cover classes were classified as “non-habitat” (we refer to this three-class classification as PMM3). Additionally, we used an alternative classification scheme, where we classified all “non-habitat” land cover classes as missing values coded as ‘NA’ (we refer to this two-class and NA classification as PMM2). Using R 3.6 (R Core Team, 2019) and the “landscapemetrics” package (Hesselbarth et al., 2019), we calculated 28 landscape-level metrics for each of the 136 ellipsoids, thus describing the composition, configuration and diversity of land cover classes between any pair of sites. We removed two metrics (patch richness and relative patch richness) because their values did not vary among ellipsoids, i.e., all classes were present in all ellipsoids. For the approach classifying non-habitat as NA values (PMM2), the interspersion and juxtaposition index (iji) was also removed because this metric is not defined for landscapes with less than three land cover classes. Subsequently, we corrected for metric correlation by removing metrics one after another, starting with metrics with the highest variance inflation factor (VIF), until all metrics had a VIF < 10. This resulted in 7 included metrics with PMM3, and 8 with PMM2 (Table 2).

**TABLE 2:** Landscape metrics used within the context of the patch mosaic model (PMM) to describe the heterogeneity of the intervening landscape between longhorn beetle sampling sites.

**Metric**	**Abbreviation**	**Aspect of landscape described**	**PMM3**	**PMM2**
Patch density	*pd*	Landscape fragmentation	X	X
Interspersion and juxtaposition index	*iji*	Intermixing of land cover classes	X	
Patch richness density	*prd*	Diversity of land cover classes	X	X
Mean core area	*core_mn*	Shape and area of patches	X	
Mean core area index	*cai_mn*	Ratio between patch and core area	X	X
Splitting index	*split*	Aggregation of patches	X	X
Mesh index	*mesh*	Measure of patch structure	X	X
Mean patch area	*area_mn*	Landscape composition		X
Number of patches	*np*	Landscape fragmentation		X
Edge density	*ed*	Landscape configuration		X

**The symbol ‘X’ indicates metrics included in the analysis, separately for the classifications with three classes (PMM3; treating non-habitat as a class) and two classes (PMM2; treating non-habitat as missing values, NA).**

### Resistance-Based Modeling

We used LCP and CD as descriptors within our RBM to evaluate landscape connectivity between sampling sites. We used the HSM to calculate a conductance surface, *C* (Eq. 1).

$$C=1+H^{3}\,\left(\mathrm{modified}\,\mathrm{from}\,\text{ Dickson et al., 2017}\right)$$

where *C* is the conductance and *H* is the habitat quality value taken from the HSM. This transformation was applied to emphasize high habitat quality values and ensure that conductance in the circuit was large enough to separate between high current flow compared to low current flow or bottlenecks in the current density map (Koen et al., 2014). Note that Eq. 1 results in small contrast between the high and intermediate values of habitat suitability, and high contrasts between intermediate and low values of habitat suitability, as recommended by Keeley et al. (2016).

**FIGURE 4:**
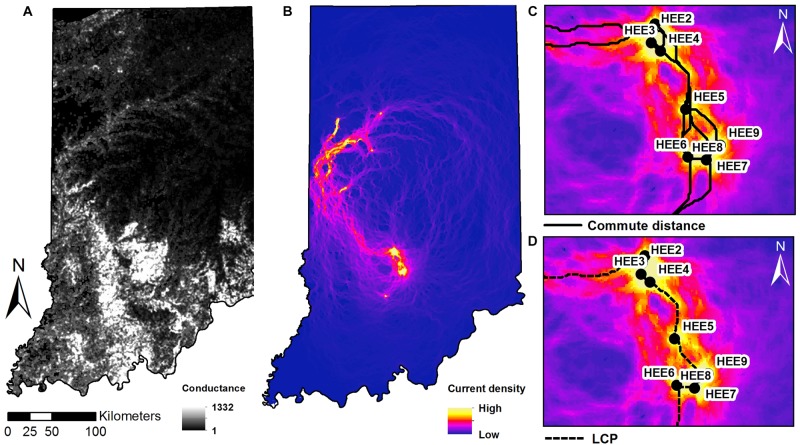
Maps of **(A)** conductance surface across the state of Indiana. **(B)** Current density map with sampling sites as nodes, and zoomed-in extent of the southern study sites in Indiana showing: **(C)** shortest paths of multiple corridors connecting between nodes and **(D)** the least cost paths plotted on the current density map.

Least-cost paths model the “expense” of an individual traveling between any pair of sites as the maximum cumulative sum of the conductance values *C*. Commute distances (van Etten, 2017) are calculated as the expected random-walk commute time between two sites. Both metrics were calculated using the “gdistance” package (van Etten, 2017) based on the HSM value (Figure 4).

Lastly, we considered a model of isolation by distance (IBD) as a null model to compare to the above-mentioned alternative approaches for quantifying landscape connectivity. This IBD model used pairwise Euclidean distances between sampling sites as a single predictor.

### Statistical Modeling

We generated MLPE models using pairwise *R*_ST_ as a response variable and patch metrics (PMM3 or PMM2), surface metrics, LCP, CD, or Euclidean distance as explanatory variables. MLPE models (Clarke et al., 2002) include two random effects for each pairwise distance, one for each sampling location, to account for the multiple pairwise distances per site (Row et al., 2017). We used a Box–Cox transformation (Box and Cox, 1964) on the *R*_ST_ values to normalize residuals and equalize variance to ensure that we meet statistical assumptions (Osborne and Carolina, 2010). Explanatory variables met normality assumptions and were not transformed, but they were scaled prior to modeling. We conducted a test for residual spatial autocorrelation for the full MLPE model for each type of metrics (Jaffé et al., 2019) and found no significant autocorrelation. Because the same sampling sites were considered in each submodel, this satisfies the spatial independence assumption for all submodels, i.e., model selection with subsets of the predictors included in the full model. All models were fitted with the “lme4” package (Bates et al., 2015) in R.

We started with fitting a full MLPE model for each modeling approach (GSM, PMM3, PMM2, LCP, CD, and IBD) incorporating all explanatory variables per approach. We used MLPE models as Row et al. (2017) found that this method did not bias model selection toward more complex models as reported for other distance-based modeling frameworks (Franckowiak et al., 2017). To select the best model for each approach, we used model dredging, which considers all possible submodels of the full model (except for LCP, CD, and IBD where only one predictor was available). In order to ensure that model predictors were not collinear, we assessed collinearity during model selection for each model independently, requiring pairwise linear correlations *r* among predictors to be less than *r* < 0.6. This ensured a systematic selection of the best model under each paradigm by considering all possible combinations of all explanatory variables while addressing multicollinearity. The best models for the GSM and PMM approaches were selected as the model, fitted with maximum likelihood (ML), with the lowest Akaike’s Information Criterion, AIC (Burnham and Anderson, 2004).

For the best model for each approach, we calculated marginal and conditional *R*^2^ values with the ‘MuMIn’ R package (Barton, 2009). Marginal *R*^2^ values represent the amount of variation explained by the fixed effects of the model (Edwards et al., 2008), after accounting for random factors and based on models refitted with REML. A high marginal *R*^2^ indicates a higher predictive power of the fixed effects in the model, whereas conditional *R*^2^ indicates the total variance explained by fixed and random factors combined. To provide an estimate of the pairwise dependency effect within each model, we calculated Spearman’s *rho* (ρ) with the “rhoR” (Eagan et al., 2019) package in R. If *rho* was zero, we would not need to include population effects, and the closer *rho* is to 1, the more important it is to account for the pairwise dependency. All R scripts are available at https://zenodo.org/record/3369727.

## Results

The GSM models explained the pairwise genetic distances in the longhorn beetle population more effectively than any of the other approaches. The best model using surface metrics within the GSM approach included dominant texture direction (*Std*) and texture direction index (*Stdi*), with AIC = −68.8, a marginal *R*^2^ value of m.*R*^2^ = 0.08 and a conditional *R*^2^ value of c.*R*^2^ = 0.26. One explanatory variable was included in all of the top three GSM models; the texture direction index (*Stdi*). The top three GSM models had the lowest AIC among all modeling approaches (Table 3).

**TABLE 3:** Summary of the MLPE model outputs for each approach. Only the top three from each multivariate model structure are included.

**Approach**	**Explanatory variables**	**AIC**	**ΔAIC**	***w***	**m.*R*^2^**	**c.*R*^2^**	**ρ**
GSM	*R*_ST_ ∼ Std + Stdi	−68.8	0	0.030	0.08	0.26	0.49
	*R*_ST_ ∼ Ssk + Std + Stdi	−68.5	−	−	0.09	0.27	
	*R*_ST_ ∼ Sfd + Stdi	−68.4	−	−	0.08	0.25	
PMM3	*R*_ST_ ∼ split	−64.5	4.3	0.003	0.05	0.21	0.46
	*R*_ST_ ∼ mesh + split	−63.2	−	−	0.06	0.21	
	*R*_ST_ ∼ core_mn + split	−63.0	−	−	0.05	0.21	
PMM2	*R*_ST_ ∼ cai_mn + pd + prd	−64.2	4.6	0.003	0.07	0.29	0.48
	*R*_ST_ ∼ cai_mn + np + pd + prd	−63.5	−	−	0.08	0.31	
	*R*_ST_ ∼ cai_mn + ed + np + pd + prd	−63.6	−	−	0.11	0.35	
CD	*R*_ST_ ∼ CD	−61.9	6.9	0.001	0.03	0.23	0.48
IBD	*R*_ST_ ∼ euclidean distance	−60.1	8.7	0.0003	0.005	0.21	0.47
LCP	*R*_ST_ ∼ LCP	−59.8	9.0	0.0003	0.003	0.21	0.48

*GSM, gradient surface model; PMM, patch mosaic model with three (PMM3) or two classes (PMM2); CD, commute distance model; IBD, isolation by distance model; LCP, least cost patch model), sorted by the AIC value of the best model for each approach. The response variable is the pairwise genetic distance (R_ST_). For more information about the explanatory variables, see Tables 1, 2. Based on models fitted with maximum likelihood (ML): AIC, Akaike Information Criteria; ΔAIC, delta AIC; w, AIC evidence weights. Based on models fitted with restricted maximum likelihood (REML): m.R, marginal R^2^ values; c.R^2^, conditional R^2^ values; ρ, Rho, which measures the strength of the population effects.*

Models based on categorical representation of the landscape (PMM) explained the pairwise genetic distances in the longhorn beetle population slightly less effective than the GSM. The best model using landscape metrics within the PMM approach, based on three classes (PMM3; habitat, complementary habitat, and non-habitat), included only the splitting index (*split*) as explanatory variable, with AIC = −64.5, a marginal *R*^2^ value of m.*R*^2^ = 0.05 and a conditional *R*^2^ value of c.*R*^2^ = 0.21. The splitting index was included in all of the top three PMM3 models. The AIC value of the best model for the PMM approach with only two land cover classes (PMM2; treating non-habitat as missing values) was similar (AIC = −64.2, m.*R*^2^ = 0.07, c.*R*^2^ = 0.29), however, the included metrics differed. The best model included the mean core area index (*cai_mn*), patch density (*pd*), and patch richness density (*prd*) (Table 3).

For the resistance-based approaches, models were independently run with CD and LCP as the sole explanatory variables. Both of these models poorly explained the pairwise genetic differences in the beetle population. However, the CD model performed better than the LCP model as indicated by the AIC, and both R^2^ values (see Table 3).

Finally, distance alone as a predictor in the IBD model was the least effective in explaining the pairwise genetic differences in the beetle population among all other modeling approaches except for LCP (see Table 3).

## Discussion

### Landscape Modeling Paradigms in Landscape Genetic Studies

This study compared the ability of different approaches to model habitat heterogeneity and functional connectivity for the banded longhorn beetle, an arthropod that relies on the proximity of forest and open habitats. In agreement with our predictions, we found that conceptually different approaches to model landscape heterogeneity and connectivity (Figure 1) differed in their ability to explain the spatial genetic structure of this species. We found that GSM applied to a continuous HSM were the most effective at explaining pairwise genetic distances as a proxy for gene flow across the landscape, followed by landscape metrics calculated from the discrete PMM, whereas analysis of the HSM with LCP performed worse than the same analysis using CD and a null model of isolation by distance (IBD).

Modeling landscape resistance to gene flow has become a main focus of landscape genetic studies (Spear et al., 2010). While many studies consider alternative resistance values assigned to discrete landscape features, such as land-use/land-cover classes, or alternative response functions to continuous variables, such as slope (Austin, 2002), little attention has been given to comparing alternative landscape representations and their impact on our ability to model gene flow in heterogenous landscapes. Here we used the example of a species that requires landscape complementation to showcase and compare little-used alternatives that may be used to represent and integrate multiple habitat requirements. In this study we demonstrated that for banded longhorn beetles, gene flow across the landscape was best explained by a landscape modeling approach that considers gradients in habitat suitability, rather than discrete patches. Despite the large conceptual differences, however, the differences between these two models, as measured by AIC, were small, especially compared to the much lower performance of RBM and the null model of IBD. Our results also illustrate that different genetic distance metrics might influence landscape models’ structure and performance (see Supplementary Material). Thus, the choice of a reliable genetic distance metric as a proxy of gene flow in landscape genetic studies is important and has to be done with insight into relevant evolutionary models of the molecular marker(s) being used. Yet, comparing the behavior of different genetic distances in landscape models (e.g., Séré et al., 2017) remains a topic for investigation that is outside the scope of this study.

### Gradient Surface Metrics (GSM)

We found that the top three gradient surface metrics models (GSM) had the lowest AIC values overall and therefore, the GSM approach was the most effective at explaining gene flow across the landscape for banded longhorn beetles. GSM (Table 1) can provide a complex characterization of the effect of landscape heterogeneity on gene flow. This is based on two steps: building the HSM with patch-based (discrete) and gradient-based (continuous) landscape features, including landscape complementation, and evaluating them within a biologically relevant neighborhood (moving window analysis). The surface metrics then provides a suite of sophisticated measures for characterizing heterogeneity and spatial gradients in habitat suitability, including metrics that have no analogs from the PMM (McGarigal et al., 2009; Abdel Moniem and Holland, 2013). In this study, GSM models predictively outperformed PMM models. However, this increased complexity of landscape heterogeneity quantification comes at the cost of less intuitive interpretation compared to the PMM approach.

Skewness (*Ssk*) is an amplitude metric that measures whether high (peaks) or low (valleys) values of the habitat suitability surface dominate the landscape. It is an important descriptor of the degree and nature of land cover dominance in the landscape. The dominant texture direction (*Std*) and texture direction index (*Stdi*) are important configuration metrics that describe the orientation of the dominant directionality of habitat suitability surface in the landscape. These metrics can be informative if repeated changes in habitat suitability exist in a certain direction. Habitat features are driven by these landscape textures (Adra et al., 2013) and they are therefore important in species ecology and distribution, but the interpretation of these quantities may not be intuitive. The information on dominant direction of habitat suitability surface in the landscape is unique to these two GSM metrics. The surface fractal dimension (*Sfd*) is a bearing metric that describes the radial texture of the surface (angles of peaks) based on Fourier analysis (McGarigal et al., 2009). Biologically, this could be interpreted as the degree of complexity of high habitat suitability (surface peaks) spread measured from the center of a given landscape. Collectively, the amplitude, configuration, and bearing metrics of GSM are very powerful descriptors of both spatial and non-spatial aspects of heterogeneity in the landscape. Yet, the biological relevance of these GSM metrics is not necessarily easy to conceptually interpret at the landscape scale and more research on the behavior of these metrics is required, especially in a landscape genetics context to further understand their biological and ecological relevance.

One metric, texture direction index (*Stdi*), was included in the top-three GSM models. The texture direction index describes the spatial structure of the HSM surface as a proportion of the dominant direction of surface roughness, which is indicative of changes in habitat suitability in different compass directions. Although this variable predominantly explains landscape texture, not habitat, the two are routinely interrelated (e.g., Adra et al., 2013).

This study used the same HSM as Abdel Moniem et al. (2016), which integrated multiple discrete and continuous habitat characteristics in a moving-window analysis. While this model explicitly considered percent forest, the availability of complementary, open habitat was not quantified directly, but subsumed in the landscape splitting index (McGarigal et al., 2009) which considers all cover types simultaneously. It is possible that a more explicit quantification of the availability and adjacency of forest and open habitat could further improve the performance of our GSM models.

### Landscape Metrics and the PMM

The best PMM models performed almost equally as well as the best GSM models. Despite a lower model AIC score, the PMM may have an interpretational advantage over the GSM. The biological meaning of specific landscape metrics in the PMM approach (e.g., intermixing of land cover classes) is easier to interpret than the properties of a habitat suitability surface represented by GSM in a GSM approach (e.g., surface roughness). For instance, the top three PMM3 models all included the splitting index. Note that for the PMM3 model, the index was calculated as a single value for each ellipsoid, whereas for the HSM used above, a separate value of the index was calculated within a moving window centered around each grid cell, and the gradient in the local aggregation of patches measured by this index surface was combined with other variables into the HSM. The GSM then characterized the properties of this combined surface. In contrast, the interpretation of a single value for the splitting index for PMM3 per ellipsoid is much more direct and intuitive.

Models using discrete landscape metrics may be valuable for species that require landscape complementation, especially in landscapes dominated by human influence that are quite well described by a patch mosaic. Interestingly, AIC values were similar between a classification scheme in which non-habitat for the banded longhorn beetle was classified as a third cover class (PMM3) compared to a classification scheme in which non-habitat was classified as missing values (PMM2). The latter resulted in a considerably higher explanatory power (values for marginal and conditional *R*^2^). However, the top PMM2 models also had more predictors than the top PMM3 model, resulting in the best-ranked PMM model being PMM3 with a single predictor. This may suggest that the PMM approach is quite robust toward the definition of land cover classes, or that neither approach is optimal.

Historically, landscape metrics (reviewed in McGarigal et al., 2002; Uuemaa et al., 2009) have been developed for a single cover type (class-level metrics) or for the mosaic of all cover types present in a map (landscape-level metrics), but not for a combination of two cover types (complementary habitat types, such as forest and open land) embedded among other types (non-habitat). This may have led to a trade-off between our PMM3 and PMM2 models, where neither is optimally suited to capture landscape complementation. For instance, the interspersion and juxtaposition index measure the “intermixing of land cover classes” (Table 2; McGarigal et al., 2002; Hesselbarth et al., 2019) and thus, biologically, seems ideal for quantifying the proximity of forest and open cover types, the two complementary habitat types used by banded longhorn beetles. In the PMM3 model, however, the index does not distinguish between the interspersion of forest with open habitat and of forest with non-habitat or open habitat with non-habitat. It is logical to address this by coding non-habitat as missing values, however, then the index can no longer be calculated. This highlights the need for developing landscape metrics that can specifically address landscape complementation.

### Resistance-Based Models (LCP and CD)

The RBM (LCP and CD) performed much worse than the GSM and PMM models. This seems intuitive since RBM reduce all information describing habitat heterogeneity and landscape complexity into a single metric (LCP or CD). Thus, these models might be suboptimal for describing landscape complementation in complex landscape compared to the GSM and the PMM. LCP and CD models are routinely and successfully applied to modeling gene flow across heterogeneous landscapes (e.g., Wang et al., 2009). While the use of a HSM to derive a resistance or conductance surface is not new (McRae, 2006; McRae et al., 2008), it differs from the more commonly used assignment of resistance values to landscape features, such as discrete cover types, and we cannot exclude the possibility that a different assignment of resistance values would improve the performance of LCP or CD models. It is also important to realize that habitat suitability does not always indicate the degree of landscape permeability to dispersal because the movement of organisms in the landscape is different than habitat selection (Mateo-Sánchez et al., 2015). Therefore, incorporating reciprocal causal models in a multi-model framework rather than just direct conversion of habitat suitability values may be warranted. Strikingly, CD explained 3% of genetic variation in banded longhorn beetles, which is considerably less than the best GSM or PMM models but much better than the LCP or the null model of IBD, which explained 0.3 and 0.5%, respectively. The genetic signal explained by CD suggests that the HSM had some validity as a representation of resistance to gene flow in banded longhorn beetles. Future research should explore to what degree the relatively low performance of LCP or CD, as applied to a HSM based on moving window analysis, can be generalized to other systems. Further research should also test for the effect of alternative scaling, e.g., by using a genetic algorithm to optimize the scaling function used to translate habitat suitability values into conductance or resistance values (e.g., “ResistanceGA”; Peterman, 2018).

The poor performance of the IBD model, which does not rely on assumptions about resistance values, suggests that in this system, environmental heterogeneity is more important than distance alone in explaining gene flow, and that ‘ecological distance’ (or isolation by resistance, IBR) as experienced by banded longhorn beetles is not confounded with geographic distance (IBD). Note that we did not consider an isolation by environment (IBE) model in this study as we focused on the quantification of habitat heterogeneity of the intervening landscape between sites. In contrast, IBE models focus more on site specific environmental and ecological descriptors to model genetic structure independently from geographic distance or the nature of the intervening landscapes between sites (Wang and Bradburd, 2014).

### Anthropocene

Anthropogenic landscape change directly impacts the structure and function of natural ecosystems (DeFries et al., 2004; Lee et al., 2006; Hobbs et al., 2009). For example, some landscapes have been delimited into grid-like blocks with sharp edges resulting in losses of natural habitat, and biodiversity (Ellis et al., 2012). Therefore, in light of the rapid progression of contemporary landscape change, it has become essential to identify approaches that quantify spatial heterogeneity in biologically meaningful ways. Relevant and comprehensive descriptors of landscape heterogeneity should consider both the landscape in context and the focal species’ requirements. As our study demonstrates, for species with complex biological requirements such as landscape complementation, metrics based on gradient surface models may be better able to capture pertinent landscape features. As technology, access to landscape data, and the resolution of genetic data, continues to improve it seems plausible that gradient surface models will continue to outperform PMMs or RBM especially in complex heterogenous landscapes. However, other modeling approaches might be more relevant when human footprints lead to patchy landscapes with more features and sharp edges (e.g., in agroecosystems: Weibull et al., 2003; Mandelik et al., 2012). In the interim, case studies, such as the one presented here, continue to be important for understanding and describing complex landscape heterogeneity, and for testing the efficacy of species-specific models for landscape genetics analyses. Our findings can inform future studies that seek to model habitat connectivity in complex heterogeneous landscapes as natural habitats continue to become more fragmented in the Anthropocene.

## Data Availability Statement

Sampled landscapes, surface metrics, and microsatellite genotype datasets analyzed during the current study are available in the Dryad repository http://datadryad.org/review?doi=doi:10.5061/dryad.g5065. Additionally, all R scripts and PMM data for the statistical analysis can be found at https://zenodo.org/record/3369727.

## Author Contributions

HA and HW conceived the study and advised the international team of graduate students involved in this project. HA collected the specimens and managed the original GIS data. HA performed the molecular lab work. RB and MH carried out the GIS and statistical analyses with guidance from HA and HW. AP, AF, RB, and MH drafted the first version of the manuscript with HA and HW contributing sections, and all authors contributed to edits and manuscript revisions. The author sequence is alphabetical with exception of the advisors, where HA acted as the senior author.

## Conflict of Interest

The authors declare that the research was conducted in the absence of any commercial or financial relationships that could be construed as a potential conflict of interest.

## Abbreviations

AIC: Akaike’s Information Criterion
CD: commute distance
GSM: gradient surface metrics
HSM: habitat suitability model
IBD: isolation-by-distance
LCP: least cost path
MLPE: maximum likelihood population effects
NDVI: normalized difference vegetation index
NLCD: National Land Cover Dataset
PMM: patch mosaic model
RBM: resistance-based models
REML: restricted maximum likelihood.
