# Supplementary material for: Alternative Quantifications of Landscape Complementation to Model Gene Flow in Banded Longhorn Beetles [Typocerus v. velutinus (Olivier)]
Source: Front Genet. 2020 Mar 31;11:307. doi: 10.3389/fgene.2020.00307 (PMC7136975; doi:10.3389/fgene.2020.00307)
Supplement: Supplementary file 1 [file Data_Sheet_1.PDF]

Supplementary Material 1: Proportion of Shared Alleles (D_{PS}) Results

1 Supplementary Table 1

Table S1. Summary of the three highest-ranking MLPE models for each approach (PMM = patch mosaic model with two (PMM2) or three classes (PMM3), GSM = gradient surface model, CD = commute distance model, LCP = least cost path model, and IBD = isolation by distance model), sorted by the AIC value of the best model for each approach. The response variable is the *proportion of allelic frequency* (D_{PS}). For more information about the explanatory variables, see Tables 1 & 2 of the main text. Based on models fitted with maximum likelihood (ML): AIC = Akaike Information Criteria, ΔAIC = delta AIC, w = AIC evidence weights. Based on models fitted with restricted maximum likelihood (REML): m.R^2 = marginal R^2 values, c.R^2 = conditional R^2 values, ρ = Rho, which measures the strength of the population effects

Approach	Explanatory variables	AIC	ΔAIC	W	m.R^2	c.R^2	ρ
PMM2	D_{PS} ~ cai_mn	-345	0	0.059	0.04	0.08	0.47
	D_{PS} ~ cai_mn + split	-344	-	-	0.05	0.09	
	D_{PS} ~ cai_mn + np	-344	-	-	0.05	0.09	
PMM3	D_{PS} ~ cai_mn	-344	0.86	0.038	0.04	0.08	0.47
	D_{PS} ~ core_mn + iji	-344	-	-	0.05	0.09	
	D_{PS} ~ core_mn	-343	-	-	0.02	0.09	
GSM	D_{PS} ~ FULL†	-342	2.57	0.016	0	0.04	0.48
	D_{PS} ~ Ssk	-341	-	-	0.009	0.06	
	D_{PS} ~ Sa	-341	-	-	0.008	0.05	
CD	D_{PS} ~ CD	-341	4.39	0.007	0.002	0.04	0.50
LCP	D_{PS} ~ LCP	-340	4.51	0.006	0.001	0.04	0.46
IBD	D_{PS} ~ Euclidean distance	-340	4.68	0.006	<0.001	0.04	0.46

† FULL: Refers to all GSM explanatory variables in the full model: D_{PS} ~ *Sa + S10 + Ssk + Sdr + Std + Stdi + Srwi + Sfd + Sbi*.
